# A Review of Heavy Metal Concentration and Potential Health Implications of Beverages Consumed in Nigeria

**DOI:** 10.3390/toxics5010001

**Published:** 2016-12-22

**Authors:** Sylvester Chibueze Izah, Iniobong Reuben Inyang, Tariwari C. N. Angaye, Ifeoma Peace Okowa

**Affiliations:** Department of Biological Sciences, Faculty of Science, Niger Delta University, Wilberforce Island, Yenagoa P.M.B. 071, Bayelsa State, Nigeria; dr.inyang2009@gmail.com (I.R.I.); maktarry@yahoo.com (T.C.N.A.); ifeomapeaceokowa@gmail.com (I.P.O.)

**Keywords:** alcohol, beverages, contamination, health impacts, heavy metals, soft drink

## Abstract

Beverages are consumed in Nigeria irrespective of age, sex, and socioeconomic status. Beverages may be alcoholic (wine, spirits, and beers) or non-alcoholic (soft drink, energy drinks, candies, chocolates, milks). Notwithstanding, most beverages are packed in cans, bottles, and plastics. This paper reviews the concentration of heavy metals from some commercially-packaged beverages consumed in Nigeria. The study found that heavy metal concentrations, including iron, mercury, tin, antimony, cadmium, zinc, copper, chromium, lead, and manganese, seldom exceed the maximum contaminant level recommended by the Standard Organization of Nigeria (SON) and the World Health Organization (WHO) as applicable to drinking water resources. The occurrence of heavy metals in the beverages could have resulted from the feedstocks and water used in their production. Consumption of beverages high in heavy metal could be toxic and cause adverse effect to human health, depending on the rate of exposure and accumulation dosage. This study concludes by suggesting that heavy metal concentration in the feedstocks and water should be monitored by producers, and its concentration in beverages should also be monitored by appropriate regulatory agencies.

## 1. Introduction

Food is a vital substance required by all organisms for the sustenance of life, and its associated functions, such as growth, development, and maintenance of the body [1,2,3]. Most food materials are mainly derived from plants (fruits, vegetables, cereals, tuber, grains, etc.) and animals [3]. Food provides the body with essential resources, such as vitamins and minerals. Foods are typically classified according to readiness and convenience to consumption. Some are consumed without further preparation after purchase (i.e., ready-to-eat food) and the category that requires further processing before consumption. Iwegbue et al. [4] described ready-to-eat food as food that is ready for consumption at the selling point. Of recent, the consumption of ready-to-eat food has increased [5]. Iwegbue et al. [4] attributed the increase in the consumption of ready-to-eat food to increased mobility, itinerary workers, and low home-centered activities. Some of the notable ready-to-eat food include fruits, fruit juices, nutritional drinks, snacks [5], and beverages [6].

Beverages consumed in Nigeria are produced locally or imported. Some local beverages widely consumed in Nigeria include palm wine (produced from the Raphia palm and oil palm tree) [7,8], zobo (a food-drink produced from the leaves of *Hibiscus sabdariffa*) [9], kunu (produced from cereal, such as sorghum (*Sorghum bicolor*), millet (*Penisetum typhoides*), maize (*Zea mays*), rice (*Oryza sativa*), wheat (*Triticum aestivum*), and acha (*Digitalis exilis*) [6,10,11,12]. Typically, non-alcoholic beverages, such as zobo and kunu, are vended in public places, including markets, motor parks, streets, outside schools, hospitals, and even expressways [2,5,6]. Generally, beverages are sold in restaurants, bars, supermarkets, and shops, or served at social functions, such as festivals, funerals, naming, weddings and marriage ceremonies. Beverages are also consumed at home by families during relaxation. However, the choice of beverages depends on individuals and locality.

Heavy metals are naturally-occurring elements that have high atomic numbers and densities higher than the density of water by at least five times [13,14]. Most heavy metals occur in the Earth’s crust. Heavy metals cannot be easily degraded [15]. Heavy metals are found in a diversity of food materials, including tea [16], fisheries [17,18,19,20,21,22], food condiments [23], ready-to-eat foods, such as beans, cake, egg rolls, hot dogs, queen cake, pudding, burgers, sausage rolls, puff-puff, buns, doughnuts, chin-chin, fish pies, meat pies, bread and spongy cake [4], fried yams, fried bean cake, roasted plantain, meat (suya) and fish, cassava flour, yam flour, garri, beans, herbal drinks (agbo jedi jedi) [1], fruits, such as apples, pineapples, oranges, watermelons, bananas, and oranges (nickel) [24], vegetables, such as fluted pumpkins [25], and beverages [26,27,28].

The occurrence of heavy metals in food often results from environmental and industrial contamination. Nnorom et al. [23] linked heavy metal contamination in food to environmental pollution. Others routes through which humans are exposed to heavy metals include contaminated air, water, and soil [29]. It could also be from the raw materials used for the processing of the specific food products and water. 

The production of beverages typically employs plant material as an active ingredient. For instance, during the production of beers, hops and cereals are used, which are plant-based and the soil in which they are cultivated could be contaminated by pesticides and fertilizers, which may contain traces of heavy metal [27]. They could also be contaminated during processing and packaging [30].

Heavy metals are classified as essential and non-essential heavy metals. Essential heavy metals and their roles have been recently documented by Izah et al. [14]. The essential heavy metals are beneficial to human health and other living things. However, essential heavy metals can be toxic to living things when the concentration exceeds the tolerable limit for the organisms. Non-essential heavy metals could be toxic to cells of the body even at low concentrations.

Heavy metals could build up in different body parts of humans, including blood, kidney, liver, heart, and spleen, where they cause disease conditions [31]. Due to the toxicity of heavy metals, their availability in food and drink is of immense concern to public health due to their biotoxic effects. Ochu et al. [32] reported that biotoxic effects of heavy metals occur when the concentration exceeds bio-recommended levels.

Due to the fact that some heavy metals have the tendency to cause irreparable damage to the human body, their concentration in ingested beverages need to the monitored. Several heavy metals, including mercury, lead, zinc, cadmium, iron, manganese, chromium, antimony, tin, copper, nickel, and arsenic, have been reported in beverages consumed in Nigeria. Among the heavy metals lead, cadmium, mercury, and arsenic are the main threats to human health on exposure [33]. Heavy metal concentration often exceeds the concentration in potable water resources [14,34], and fisheries from surface water in Nigeria [17]. Typically, humans absorb heavy metals through drinking water, food, and air [35]. Long-term exposure could slowly lead to progressing physical, muscular, and neurologically-degenerating disease conditions [35].

The assessment of heavy metals in beverages sold and consumed among a large number of people is an essential issue for consumer safety [30]. Orisakwe and Ajaezi [36] noted that the increased public concern toward food hazards and the decrease in food risk regulators suggests the need to develop effective and efficient risk communication channels in the food industry. Therefore, this study assesses the potential heavy metal toxicity associated with beverage consumption in Nigeria.

## 2. Methodology

In this study secondary data from Internet sources was used. Literature reports on the concentration of heavy metals in alcoholic and non-alcoholic beverages consumed in Nigeria between 2007 and 2016 was assessed. The mean and/or range data was extracted and presented in Table 1 and Table 2 in Section 4. The concentration of heavy metals in beverages is usually assessed based on drinking water guidelines [30]. As such, the concentrations were discussed based on limits of the Standard Organization of Nigeria [37] and World Health Organization [38]. Probable health impacts associated with heavy metal concentration in beverages are discussed in brief. The study concludes by suggesting measures of improving the quality of beverages consumed in Nigeria.

## 3. Diversity of Beverages Consumed in Nigeria

Several beverages are consumed in Nigeria, including alcoholic i.e., wines and beer, and non-alcoholic. i.e., soft drinks and some wine. Each class of beverage that is sold in supermarkets and major outlets are packaged in metallic plastics and bottles. This section of the paper focuses on the type of beverages consumed in Nigeria.

### 3.1. Non-Alcoholic

Non-alcoholic beverages are drinks whose alcoholic content is <0.5%. This is because in some popular soft drinks sold in Nigeria alcohol has been reported via a qualitative approach. Additionally, classification of beverages with regard to alcoholic content differs from country to country. For instance, Engwa et al. [39] reported that bottled, canned, and plastic drinks like Sprite, Fanta, Coke, Pepsi, Miranda, as well as sachet beverages like Don Simon pineapple juice, Chi Active 5, citrus fruit juice, fresh pineapple and coconut juice contain alcohol, while bottled Maltina, Amstel, 7Up, canned Amstel, Maltina, plastic-bottled 7Up, Chivita premium pineapple and coconut juice and fresh citrus juice do not contain alcohol. Additionally, some of the non-alcoholic beverages consumed in Nigeria are imported. For instance Maduabuchi et al. [40] reported the arsenic and chromium levels of some canned beverages consumed in Nigeria and their place of manufacture include Picnic Soymilk (Maeil), produced in Seoul, South Korea; Remmy Rankky Orange, manufactured in Wuging, Republic of China, Sprite soft drink, manufactured in Wadeville, South Africa; Star Pino Pineapple and Star Mango, produced in Shariah, United Arab Emirates; Godys Malta Drink, manufactured in Germany; and Chinchin malt milk drink, produced in Tianjin, China. Some un-canned foods, including La Casera Orange Drink, Chelsea Teezer Gin and Pinneapple, Fine Merit Yoghurt, Delite Black Currant Drink, Chivita Orange Juice, Popcy Flavored Drink, Lulu Apple Juice, produced in Lagos, Nigeria; Sans Cream Soda, Ribena Black Currant, Lucozade Boost, produced in Ogun State, Nigeria; V. Roovers Orange Drink, from Ogidi, Nigeria; Campina Yazzo Milk Drink, manufactured in Aalter, Belgium; Mighty Nice Chocolate Drink, produced in Cape Town, South Africa; Sheeza Mango, produced in Karachi, Pakistan; Vitamilk Soyamilk, produced in Thailand; and Grape Joy of Health manufactured in Cansavay Bay, Hong Kong.

Some non-alcoholic beverages are often referred to as soft drinks [30]. Soft drinks are water-based flavored drinks than can be sweetened, acidulated, or carbonated [30] and frequently colored [41]. They are mainly consumed by all sexes, ages, socioeconomic classes, and religions probably due to their affordability, taste, and thirst quenching potentials [30,41,42]. As such, they are one of the most consumed beverages in Nigeria [39]. Due to increased demand of soft drinks, the quality may be compromised [39].

Nearly all non-alcoholic drink has some level of sugar. Woyessa et al. [31] noted that the consumption of sugar-sweetened soft drinks could lead to obesity, dental caries, and low nutrient levels. The authors further asserted that sugar-sweetened drinks contain high-fructose corn syrup compared to those containing sucrose.

### 3.2. Alcoholic

Alcoholic beverages are mainly wine, spirits, and beers. Some traditionally-processed beverages, such as palm wine, are not canned and have a low shelf life. This is because as the tapping period increases the sugar content reduces and alcohol concentration increases due to the activities of indigenous microbes, such as *Saccharomyces cereivisae*. Palm wine is typically consumed by several million people in West Africa, especially in Nigeria.

In addition to palm wine, most alcoholic beverages are packaged in cans and bottles. The consumption of beer has increased within the last few decades, even in countries where they are not traditional [27]. This suggests that importation is a major business, globally. Unlike non-alcoholic beverages, alcoholic beverages are predominantly consumed by youth and the elderly. Like soft drinks, some alcoholic drinks are imported. Udota and Umoudofia [43], listed Beck (produced in Germany), Heineken (produced in Holland), and Holstein (produced in USA), Star Beer, and 33, produced in Nigeria, and Ufofop (traditional distilled gin), bull gin, and Chelsea dry gin, as commercial gins frequently consumed in Nigeria. Salako et al. [44] listed Harp, 33, Star, Gulder, Heineken, Guinness, Smirnoff, Red Bull, and Tubo as some alcoholic beverages consumed in Nigeria. Several others brand of imported and locally-brewed alcoholic drinks exist in most supermarkets, restaurants, and bars across the country. A local alcohol beverage is also brewed in Nigeria. Among the popularly-brewed beverages is “ogogoro”, which is a source of concern [45].

## 4. Heavy Metal Concentration in Food-Drink Consumed in Nigeria

The essential heavy metal is required by human at low concentration. Some notable essential heavy metals include iron, zinc, copper, chromium, cobalt, and manganese [14,46]. For instance, low concentration of heavy metal are required by the body daily includes manganese (2–5 mg/day), chromium (0.005 mg/day), cobalt (0.0001 mg/day), zinc (15–20 mg/day of which 99% is found intracellular and 1% in the plasma), iron (1–2 mg/day of which 75% is found in the blood and the rest 25% in the bone marrow, liver etc.), and copper (2–5 mg/day of which 50% is absorbed from the gastrointestinal tracks) [14,46]. Most of the heavy metals are ingested through food and water consumed by humans. In addition the consumption of alcoholic and non-alcoholic beverages is also a major route through which heavy metal enters human body.

The reference limits for heavy metal in beverages including alcoholic and non-alcoholic drinks, fruit juices, candy have been generally compared with potable water limits [26,27,30]. From drinking water limits, the United States Environmental Protection Agency evaluated the reference range of heavy metal toxicity in substances, such as beverages, i.e., soft drinks, to base a maximum contaminant level goal (MCLG), i.e., a level of contaminants in drinking water in which there is no risk to human health. As such, this allows for marginal safety and establishes a non-enforceable public goal and maximum contaminant level (MCL), i.e., the highest level of contaminant that is allowed in drinking water [31,39,41]. The MCL is further classified into two thresholds: a primary maximum contaminant level (PMCL), i.e., consideration is based on health and intended to protect people from pathogens, radioactive elements, and toxic chemicals; and a secondary maximum contaminant level (SMCL), i.e., intended for aesthetic issues, including taste, odor, or color [30]. As such, heavy metals exceeding the limits in potable water as stipulated in Nigeria water quality standards [37] and World Health Organization [38] are considered to be detrimental to human health. Table 1 and Table 2 present the concentration of heavy metals in non-alcoholic and alcoholic drinks consumed in Nigeria, respectively.

### 4.1. Iron

Iron is generally found in the environment. Its concentration in the environment varies from place to place depending on the geology of the region. Iron plays an essential role in living organisms, such as the formation of hemoglobin, transferrin, ferritin, and some iron-containing enzymes [47], metabolic processes such as oxygen transport, deoxyribonucleic acid synthesis, electron transport chain, and regulation of cell growth and differentiation [14,48,49,50], heme-moieties of hemoglobin and cytochromes [4,51]. The concentration of heavy metal found in alcoholic and non-alcoholic beverages often exceed the permissible limit of 0.3 mg/L as specified by SON [37] in drinking water. The concentration of iron in different non-alcoholic drink reported by Ogunlana et al. [15], Salako et al. [44], Adegbola et al. [52], Magomya et al. [30], and Maduabuchi et al. [29] (Table 1) often exceed the limits in some of the drinks studied. Like non-alcoholic beverages, iron concentration has been reported in alcoholic drinks, as reported by Salako et al. [44], Ubuoh [27], Udota, and Umuodofia [43] (Table 2).

### 4.2. Zinc

Zinc is one of the major essential elements required by the human system [14]. Zinc plays several functions in the human body, such as wound healing, blood clotting, proper thyroid function, maintenance of good vision [14,44,53], taste acuity, prostaglandin production, bone mineralization, cognitive functions, fetal growth, sperm production, cell growth, development, differentiation, homeostasis, connective tissue growth and maintenance, protein synthesis, DNA synthesis, RNA transcription, cell division, and cell activation [14,51,53]. Zinc supplements also reduce the duration of malaria and respiratory infections [16]. The concentration of zinc in beverages is usually within the reference range of 3 mg/L in drinking water as reported by Ogunlana et al. [15], Salako et al. [44], Iweala et al. (2014), Magomya et al. [30], and Adepoju-Bello et al. [41] (Table 1). However, few instances of heavy metals exceeding the reference range have been reported in Schappes (7.38 mg/L) by Salako et al. [44]. Similarly, the zinc concentration is below the permissible limits in some alcoholic beverages consumed in Nigeria as documented by Salako et al. [44], Iweala et al. [1], Ubuoh [27], Udota, and Umuodofia [43] (Table 2).

### 4.3. Cadmium

Cadmium is one of the toxic heavy metal to human tissues even at low concentration. Cadmium is used in several industries and they also occur naturally in the environment i.e., the Earth’s crust. Authors have reported that non-alcoholic beverages usually have low cadmium content i.e., within the permissible limits as reported by Adepoju-Bello et al. [41], Engwa et al. [39], Salako et al. [44], Magomya et al. [30], and higher concentrations above MCL limits have been reported by Iweala et al. [1] and Adegbola et al. [52]. Additionally, instances of cadmium exceeding MCL limits have been reported in bottled Coke [39], Maltina [44], and herbal drinks [1] (Table 1). Similarly low cadmium concentration have been reported in some brand of beer [27] and high concentration in alcoholic including Gulder, Heineken, Guinness, and Smirnoff [44], and herbal drinks [1] (Table 2).

### 4.4. Chromium

Chromium is one of the toxic essential heavy metals. It is highly detrimental to humans when its concentration exceeds tolerable limits by humans. It aids in the biosynthesis of glucose tolerance factor [4,14,46,51,52], utilization of sugar protein and fats [36], catabolism of fat and carbohydrates, and the maintenance of blood glucose, especially in diabetic patients [44]. In beverages consumed in Nigeria, chromium concentration below the MCL has been reported by Ogunlana et al. [15], Adegbola et al. [52]. However, higher concentrations have been reported in juice and milkshakes by Adegbola et al. [52], and other non-alcoholic beverages [30,40,41] (Table 1). However, high concentrations of chromium have been reported by Ubuoh [27], and low concentrations in some beverages have been reported by Salako et al. [44].

### 4.5. Lead

Lead is found in the Earth’s crust and has been reported to emit from anthropogenic activities, such as combustion of fossil fuels, mining, paint, batteries production, etc. Lead is found above MCL limits in beverages (alcoholic and non-alcoholic), as reported by Engwa et al. [39], Ogunlana et al. [15], Ogunlana et al. [15], Adegbola et al. [52], and Magomya et al. [30], and low concentration by Adepoju-Bello et al. [41], and Iweala et al. [1] in some non-alcoholic drinks (Table 1). Similarly, in alcoholic beverages low concentration of lead have been reported Udota and Umuodofia [43] and Iweala et al. [1], while high concentrations have been reported by Salako et al. [44], Ubuoh [27] (Table 2).

### 4.6. Mercury

Mercury is a major non-essential trace metal not needed in food. As such its presence in food suggests contamination. Mercury concentration in beverages has been scantly reported. However Robert and Orisakwe [26], and Engwa et al. [39] have reported mercury concentration above the permissible limit of 0.001 mg/L as specified by WHO and SON.

### 4.7. Copper

Copper is one of the essential heavy metals found in the environment, including water and soil. The biological functions of copper include cell metabolism, normal iron metabolism, red blood cell (hemoglobin) synthesis, connective tissue metabolism, and bone development [14,46]. Copper concentration in potable water has been reported to exceed 1 mg/L and 2 mg/L for SON and WHO limits, respectively [14]. However, in some beverage drinks consumed in Nigeria, Magomya et al. [30], Ogunlana et al. [15], and Adegbola et al. [52] reported copper concentrations within the permissible limit (Table 1). However, Salako et al. [44], Adegbola et al. [52] reported higher concentrations in some non-alcoholic drinks. Similarly low concentrations of copper have been reported in alcoholic drinks by Udota and Umuodofia [43], and Ubuoh [27]. However, instances of high concentrations of copper have been reported in ethanolic herbal drinks [1] (Table 2).

### 4.8. Nickel

Nickel is one of the trace heavy metals found in the environment. It has been reported in potable water sources in Nigeria [14]. In beverages consumed in Nigeria, nickel has been detected above the permissible limits [1,29,41,44] (Table 1). High nickel concentrations above MCL limits has been reported in some alcoholic beverages consumed in Nigeria [1,27,44] (Table 2).

### 4.9. Manganese

Manganese is one of the heavy metal needed by biological system. Manganese plays an essential role in living things, including humans, such as oxidative phosphorylation, fatty acid and cholesterol metabolism, mucopolysaccharide metabolism, and activation of some enzymes [14,46]. Higher concentrations above MCL limits have been reported in canned drinks non-alcoholic consumed in Nigeria [29] and not reported in herbal drinks (non-alcoholic and alcoholic) [1] (Table 1 and Table 2).

### 4.10. Arsenic

Arsenic is one of the non-essential heavy metals found in the environment. Its concentration in ingestible items suggests contamination. However, arsenic has been reported in potable water resources in Nigeria [34]. In beverages consumed in Nigeria, arsenic has been detected above the permissible limit of 0.01 mg/L [40,44]. However, it has not been detected in some non-alcoholic drinks as reported by Ogunlana et al. [20] (Table 1) and alcoholic drinks [44] (Table 2).

### 4.11. Antimony, Tin and Silver

Antimony and tin are heavy metals that are not often studied in beverages. Antimony has no biological functions [26]. Robert and Orisakwe [26] have reported antimony and tin concentration below WHO permissible limit in beverages. Similarly Eno-Obong and Ukoha [54] have reported the occurrence of tin in some canned beverages (Olympic and Three Crown milk) and canned soft drink (maltina, Fanta, lucozade boost, Chivita pineapple) and beverages (peak, milo and five alive juice drink) consumed in Nigeria (Table 1). Similarly in a study by Adepoju-Bello et al. [41], silver was not detected in soft drinks consumed in Nigeria. Generally, antimony, tin and silver are scantly reported in food and potable water resources in Nigeria.

Furthermore, studies have summarized that most of the non-alcoholic beverages drink sold and consumed in Nigeria often exceed the desirable limits for heavy metals. The occurrence of heavy metal above recommended limit in beverages could be due to production. According to Lachenmeier et al. [55], occurrence of toxic heavy metals (lead, arsenic, antimony, cadmium, copper and zinc) could be due to unrecorded alcohol due to deficiencies in production techniques [55]. Maduabuchi et al. [29] reported that among 21 canned and 30 non canned beverages, 95.25% and 75.86% respectively (iron), 42.86% and 51.72% respectively (manganese), 80.95% and 72.41% respectively (nickel) exceeded the MCL desirable concentration. Magomya et al. [30] reported that out of 24 soft drinks, 53.33%, 7.14%, 20.83%, 29.17% and 16.67% exceeded the set safe limits for iron, copper, chromium, lead and cadmium respectively. Orisakwe and Ajaezi [36] reported that out of 30 energy drinks consumed in Nigeria (locally manufactured and imported), 66.7%, 36.7%, 70% often exceed the set limit of WHO for lead, chromium and cobalt respectively. The authors further reported that 33.3% of the energy drinks has negligible level of daily intake for lead. Ogunlana et al. [15] reported that 70% of 6 heavy metals (arsenic, cadmium, lead, zinc, copper and iron) has their concentration exceeding WHO permissible limits in 10 different brands of soft drink consumed in Nigeria. Roberts and Orisakwe [26] reported that among 38 fruits juice and soft drinks consumed in Nigeria, 0%, 86.5% and 89.2% for antimony, tin and mercury exceeds the desirable limits set by WHO. Maduabuchi et al. [40] reported that among 21 canned and 30 non canned beverages, 33.3% and 55.2% respectively (arsenic), 68.9% and 76.2% respectively (chromium) exceeded the United states Environmental Protection Agency MCL desirable concentration. As such heavy metal occurrences in beverages consumed in Nigeria need to be monitored.

Due to high concentration found in most beverages especially the non-alcoholic, the habitual intake of beverages drink especially during the dry season could lead to heavy metal toxicity in body especially in the elderly, children and pregnant women [20].

## 5. Potential Health Implications in Beverages Containing Heavy Metals

Beverages are consumed in Nigeria irrespective of age, sex, and socioeconomics. Beverages play an essential role in supporting life and have the ability to cause disease conditions when they are contaminated and/or have expired. Heavy metals have been reported in alcoholic and non-alcoholic beverages consumed in Nigeria. When their concentration exceeds the permissible limit for each of the heavy metal they become detrimental to human health.

Several diseases have been associated to heavy metals. In Nigeria, recent environmental studies have been documented in possible heavy metal diseases associated with the consumption of potable water with high heavy metal concentrations [14] and fishes from Nigeria surface water [17]. Typically, all heavy metal-related diseases are basically similar irrespective of the source of the heavy metals provided their exposure period is long enough to cause disease. Slight variations in diseases caused also exist within the oxides of the heavy metals and its forms i.e., organic and inorganic. As such, this section of the paper focuses on the diseases associated with heavy metal concentrations in alcoholic and non-alcoholic beverages sold and consumed in Nigeria.

Heavy metals have been reported to cause several types of diseases in the human body and cause damage to organs, such as kidney, liver, and bones. Heavy metals have the tendency to alter different systems in humans, including respiratory, endocrine, nervous systems, skin, blood, colon etc. Excessive heavy metals in the body could lead to toxicity or deficiency of other trace metals in the body [43]. For instance, Iwegbue et al. [4] opined that cadmium has the tendency to substitute copper and iron in cytoplasmic and membrane proteins. Excessive absorption of zinc could hinder copper and iron absorption [1]. Lanre-Iyanda and Adekunle [56] reported that cadmium could interfere with zinc and inhibit its enzymatic and nutrient utilization. The environmental occurrence, industrial production and uses, molecular mechanisms of toxicity, and carcinogenicity for some of these metals, including arsenic, cadmium, chromium, lead, and mercury, have been documented by Tchounwou et al. [13] and the occurrence, exposure, and dose in mercury, lead, arsenic, and cadmium have also been documented by Jarup [33]. Some heavy metals of particular concern in relation to human health include mercury, lead, arsenic, tin, cadmium, etc. [57].

Heavy metals, such as chromium, can cause a reduction in glucose tolerance factor and increases cardiovascular diseases [52]. High doses of cadmium could lead to liver, kidney, and bone disease conditions [14,58,59]. Cadmium has been linked to several disease conditions including skeletal, and kidney damage [4,33,57], gastro-intestinal irritation, and pulmonary effects [13], vomitting, lung damage, and even death [16].

Copper is one the essential heavy metals that cannot be formed in human body [44], but is required for physical and mental development [56]. It is usually ingested from food and fluids during consumption. Copper concentration exceeding 5.0 mg/kg body weight is toxic to humans, especially to the gastrointestinal system [44]. The major organs that excess copper can damage includes the liver and kidneys. Some of the observable symptoms associated with copper toxicity in humans include vomiting, hypertension, and gastrointestinal distress. However, the deficiency of copper could lead to hypertension, hypercholesterolemia, and increased low density lipoproteins [4].

Zinc is one of the essential heavy metals. Its deficiency resulting from diet, alcoholism, and mal-absorption could lead to dwarfism, hypogonadism, and dermatitis, while excess concentration in the body could lead to nausea, anemia, and electrolyte imbalance [31,60], and increased prevalence of obesity [61]. Excess concentration could also be detrimental to body tissues/organs.

Deficiency of iron can cause fatigue, shortness of breath, and difficulty in thinking. In beverages consumed in Nigeria high iron content has been reported. This could cause health-related problems, including liver damage [44]. Other iron-related disease conditions include lung disease and siderosis [14,59].

A high concentration of nickel has been reported in beverages. Such consumption of these beverages over a long period of time could lead to nickel-oriented disease conditions that usually affect the kidney, bones, thyroid glands [44]. The role of nickel in the human body is not fully understood [56]. Some of the symptoms associated with diseases from excess nickel exposure include nausea, vomiting, stomach upset, virtual disturbances, headache, giddiness, and cough [36]. Nickel could also inhibit DNA repair enzymes thereby producing oxygen free radicals which degrade in situ proteins [36].

Arsenic usually causes disease conditions even at low concentration. The type of disease condition caused depends on the oxide and sources. In organic sources (sources through which it is mainly contacted from beverages), arsenic can cause nerve injury and stomach aches. Izah and Srivastav [34], Tchounwou et al. [13], and Maduabuchi et al. [40] reported that high arsenic exposure could lead to cardiovascular, hematological, neurological, respiratory, gastrointestinal, and developmental disorders, dermatitis and cancer, diabetes, and hearing loss. As such, high concentrations of arsenic could affect kidney, liver, bladder, and skin [13,33]. In a few instances, arsenic exceeded the permissible limit of 0.01 mg/L. Inorganic and semi-metal arsenic compound are carcinogenic [62]. Specifically, long-term, low-dose exposure to inorganic arsenic compounds could lead to mutagenesis [62].

Lead is known to cause toxicity and poisoning in individuals exposed to them. Lead poisoning can lead to various disease symptoms, including anemia, convulsion, central-nervous system disorders [16], anorexia, headache, malaise, diarrhea, lead-palsy, encephalopathy, insomnia, [58], high blood pressure, anemia, and weakness in fingers, wrists, or ankles [59], hair loss, lung fibrosis, skin allergies, outbreak of eczema, and variable degrees of kidney operation [63], loss of appetite, vomiting, irritability and behavioral changes [1,33]. Some of the notable damage caused by high exposure to lead include kidney, liver, and brain (cerebral edema), reproductive, cardiovascular and nervous system, gastrointestinal, and neuromuscular disorders [14,57]. Furthermore, through respiratory and digestive systems, lead enters the blood and over 90% is bioconcentrated in the bone, where it is stored in the body [16].

Mercury is another toxic heavy metal that causes toxicity even at low concentration. Mercury toxicity has the tendency to interfere with several cellular metabolisms in human and can affect children and adults, depending on the exposure and concentration. Some of the symptoms associated with mercury toxicity in humans include irritability, shyness, tremors, changes in vision or hearing, and memory problems, nausea, vomiting, diarrhea, increased blood pressure, skin rashes, and eye irritation [14,33,59].

Manganese is needed by the body tissues at low concentration. Deficiency of manganese could lead to osteoporosis, epilepsy, and diabetes mellitus, and concentration above threshold values causes manganese toxicity in the body [4]. Neurological toxicity associated with excess manganese could lead to behavioral changes, which are characterized by slow movement, tremors, facial muscle spasms, irritability, aggressiveness, and hallucinations [1].

Actue and chronic toxicity of chromium occurs depending on the level of exposure and the occurring form of the element. Typically, chronic forms of chromium are characterized by eczematous dermatitis and acute toxicity, which could lead to death [40]. Others organs significantly affected by chromium toxicity include kidney, skin, neurons, and liver [13,36,59]. Some of the symptoms associated with chromium toxicity include breathing difficulty, nose problems, such as irritation and ulcers, asthma, and cough [36,59].

Antimony can cause headaches, dizziness, and depression at low concentration and vomiting at high concentration when ingested [26]. Similarly, tin could cause headaches and stomach upset leading to diarrhea. Like other heavy metals, exposure to silver over a long period of time could lead to eye, mouth, and skin staining [64]. The concentration of tin and antimony reported in this study is too low to cause disease condition.

## 6. Conclusions and Way Forward

Beverage consumption has increased in recent years. Beverages are consumed in several parts of the world, irrespective of sex, socioeconomic status, and race. Beverage drinks are consumed during social gatherings and relaxation. Nigeria is among the nations that consume a high quantity of beverages. Several non-alcoholic drinks i.e., soft drinks and energy drinks, which are predominantly packaged in cans, bottles, and plastics, abound in Nigeria. Like non-alcoholic drinks, alcoholic drinks are also consumed in large amounts in Nigeria. Heavy metals are found in the drinks at varying concentrations. These heavy metals find their way into the drinks through the raw materials used for the beverage drink production, water, and other spices. The concentration of heavy metals, such as mercury, arsenic, antimony, tin, lead, chromium, cadmium, manganese, and iron found in most beverages consumed in Nigeria often exceed MCL limits based on WHO and SON drinking water guidelines in some instances. The occurrence of heavy metals above MCL limits could lead to some disease conditions over a long period of time.

As such, producers of beverages should pay attention to the feedstocks and water used in processing with regard to heavy metal concentration in feedstocks used for processing. The packaging materials, especially canned drinks, should be investigated for possible reductions in heavy metal constituents, such as tin. Effort should be made by agencies, such as the National Agency for Food and Drug Administration and Control, in monitoring the concentration of heavy metals found in beverages consumed in Nigeria.

## Figures and Tables

**Table 1:** Heavy metal (mg/L) concentration in some non-alcoholic beverages consumed in Nigeria.

Non-Alcoholic Beverages	Fe	Zn	Cd	Cr	Pb	Hg	Cu	Ni	Mn	Sn	As	Ag	Sb	Reference
Ten selected soft drink	0.08–0.55	0.011–2.28	ND-0.01	-	ND-0.04	-	0.04–0.79	-	-	-	ND-0.01	-	-	[20]
Non-alcoholic (ferrous, scnappes, spirite, cocacola, Amstel, malta, Fanta, malta Guinness, Maltina)	0.572–1.734	0.001–7.38	ND-0.26	ND-0.026	ND-0.447	-	ND-3.256	ND-0.272	-	-	ND-0.141	-	-	[44]
Milk candy and fruit concentrates	0.61–1.99	-	ND-0.07	ND-0.001	ND-0.28	-	0.01–3.68	-	-	-	-	-	-	[52]
Juice and milk shake	0.50–1.88	-	0.08–0.12	0.06–0.57	0.20–1.21	-	0.01–0.03	-	-	-	-	-	-	[52]
Herbal drink (agbo jedi jedi) aqueous	-	0.46	0.59	-	ND	ND	3.55	6.38	ND	-	-	-	-	[1]
* Fruit juice and soft drink (grand malt, cocal cola, ginger, Fanta orange, spirte, Schweppes bitter lemmon, rubicon guava, fayrouz premium soft drink, caprisoone orange drink, dansa fruit drink, delite orange fruit juice, Chivita premium pinapple juice, chi exotic pineapple and coconut nectar, tropical pure fruit juice, ice tea lemon, locomalt, 5 alive berry blast, lucozade boost, ribenna blackcurrent, fruchtegut, La casera aplle drink, bobo apple milk drink, V8 splash berry blend, fuze healthy infusions, san cream soda, tango, old Jamaica ginger beer, Amstel malt, betamalt, nestle milo energy drink, robust sugar free, 7 up)	-	-	-	-	-	2.39	-	-	-	3.66	-	-	0.49	[26]
Twenty four selected soft drink	0.10–3.81	0.02–2.42	ND-0.03	ND-0.10	ND-0.05	-	0.07–2.20	-	-	-	-	-	-	[30]
Soft drinks (bottle coke, fanta, pepsi, Miranda, can coke, can zero coke, fanata, spirite, plastic Fanta, coke, sprite, pepsi, Miranda, Don simon pineapple juice, Chi active 5, citrus fruit juice, fresh pineapple and coconut juice and fresh pinapple juice, bottled Maltina, Amstel, 7 up, can Amstel, Maltina, Plastic, 7 up, Chivita premium pineapple and coconut juice and fresh citrus juice)	-	-	0.00–0.149	-	0.00–3.392	ND-11.33	-	-	-	-	-	-	-	[39]
* Canned soft drink (maltina, Fanta, lucozade boost, Chivita pineapple	-	-	-	-	-	-	-	-	-	<0.01–0.31	-	-	-	[54]
* Canned beverages (Olympic and Three Crown milk)	-	-	-	-	-	-	-	-	-	217.6–516.0	-	-	-	[54]
* Canned beverages (peak, milo and five alive juice drink)	-	-	-	-	-	-	-	-	-	<0.01	-	-	-	[54]
Twenty different brand of soft drink	-	ND	ND-0.158	ND-2.33	ND-0.002	-	-	ND-0.063	-	-	-	ND	-	[41]
Canned beverages (Picnic Soymilk, Remmy Rankky Orange, Sprite Soft Drink, Star Pino Pineapple, Star Mango, Godys Malta Drink, Chinchin malt milk drink, coca coloa, Glorietta Lemonade orange, Sagiko pink guava, original precious juice, Fanta orange, sobela mixed fruit drink, sweet heart mixed fruit, Gold quell multivitamin, luna milk, three crowns milk, peak milk, lino and top milk and Lino and Holsten malta drinks	-	-	-	0.04–0.59	-	-	-	-	-	-	0.003–0.161	-	-	[40]
La Casera Orange and apple Drink, Chelsea Teezer Gin, Pinneapple, Fine Merit Yoghurt, Delite Black Currant Drink, Chivita Orange Juice, Popcy Flavored Drink, Lulu Apple Juice, Sans Cream Soda, Ribena Black Currant, Lucozade Boost V. Roovers Orange Drink, Campina Yazzo Milk Drink, Mighty Nice Chocolate Drink, Sheeza Mango, Vitamilk Soyamilk Grape Joy of Health, tico orange cordial, marigold orange, lucomalt, vinamilk yomilk, 5-alive citrus burst juice, might nice vanilla low fat, caprisonne pineapple drink	-	-	-	0.01–0.55	-	-	-	-	-	-	0.002–0.261	-	-	[40]
Canned beverages (Picnic Soymilk, Remmy Rankky Orange, Sprite Soft Drink, Star Pino Pineapple, Star Mango, Godys Malta Drink, Chinchin malt milk drink, coca coloa, Glorietta Lemonade orange, Sagiko pink guava, original precious juice, Fanta orange, sobela mixed fruit drink, sweet heart mixed fruit, Gold quell multivitamin, luna milk, three crowns milk, peak milk, lino and top milk and Lino and Holsten malta drinks)	0.02–2.460	-	-	-	-	-	-	0.013–0.993	0.001–0.730	-	-	-	-	[29]
La Casera Orange and apple Drink, Chelsea Teezer Gin, Pinneapple, Fine Merit Yoghurt, Delite Black Currant Drink, Chivita Orange Juice, Popcy Flavored Drink, Lulu Apple Juice, Sans Cream Soda, Ribena Black Currant, Lucozade Boost V. Roovers Orange Drink, Campina Yazzo Milk Drink, Mighty Nice Chocolate Drink, Sheeza Mango, Vitamilk Soyamilk Grape Joy of Health, tico orange cordial, marigold orange,lucomalt, vinamilk yomilk, 5-alive citrus burst juice, might nice vanilla low fat, caprisonne pineapple drink	0.02–2.09	-	-	-	-	-	-	0.009–0.938	0.001–0.209	-	-	-	-	[29]
**SON**	0.3	3	0.003	0.05	0.01	0.001	1	0.02	0.2	-	0.01	-	-	[37]
**WHO**	-	-	0.003	0.05	0.01	0.001	2	0.07	-	0.002	0.01	-	0.02	[38]

* Expressed as μg/mL.

**Table 2:** Heavy metal (mg/L) concentration in some alcoholic beverages consumed in Nigeria.

Alcoholic Beverages	Fe	Zn	Cd	Cr	Pb	Cu	Ni	Mn	As	Reference
Herbal drink (agbo jedi jedi)		0.58	0.39		ND	3.12	1.53	ND		[1]
Harp, 33, star, gulder, Heineken, Guinness, Smirnoff, red bull, turbo	1.093–2.455	0.007–0.227	0.006–0.104	ND-0.002	ND-0.081	<0.001–0.671	0.184–0.273	-	ND-0.20	[44]
Canned beer (becks, Dettinger, Guinness, Stout, Heineken, Henburg, Hollandia, Olsten, Panther and Tuborg	0.23–0.56	0.08–0.15	0.003–0.008	0.17–0.34	0.023–0.045	0.04–0.08	0.04–0.10	-	-	[27]
Star, 33, Champion, becks, Heineken, Holstein	0.05–0.50	ND-0.643	-	-	ND	ND-0.10	-	-	-	[43]
Locally brewed gin “ufofp”	6.00–28.50	0.33–5.00	-	-	3.00–6.75	3.16–6.21	-	-	-	[43]
**SON**	0.3	3	0.003	0.05	0.01	1	0.02	0.2	0.01	[37]
**WHO**	-	-	0.003	0.05	0.01	2	0.07	-	0.01	[38]
